# Comparison of conventional polymerase chain reaction and routine blood smear for the detection of *Babesia canis*, *Hepatozoon canis, Ehrlichia canis*, and *Anaplasma platys* in Buriram Province, Thailand

**DOI:** 10.14202/vetworld.2019.700-705

**Published:** 2019-05-24

**Authors:** Rucksak Rucksaken, Cherdsak Maneeruttanarungroj, Thanaporn Maswanna, Metita Sussadee, Pithai Kanbutra

**Affiliations:** 1 Department of Veterinary Technology, Faculty of Veterinary Technology, Kasetsart University, Bangkok, Thailand; 2 Department of Biology, Faculty of Science, King Mongkut’s Institute of Technology Ladkrabang, Bangkok, Thailand; 3 Bioenergy Research Unit, Faculty of Science, King Mongkut’s Institute of Technology Ladkrabang, Bangkok, Thailand; 4 Scientific Instrument Center, Faculty of Science, King Mongkut’s Institute of Technology Ladkrabang, Bangkok, Thailand; 5 Veterinary Teaching Hospital, Faculty of Veterinary Medicine, Khon Kaen University, Khon Kaen, Thailand

**Keywords:** Blood parasites, dog, polymerase chain reaction, prevalence

## Abstract

**Background and Aim:**

Dog blood parasites are important tick-borne diseases causing morbidity and mortality in dogs worldwide. Four dog blood parasites species are commonly found in Thailand: *Babesia canis, Hepatozoon canis, Ehrlichia canis*, and *Anaplasma platys*. They are transmitted easily by tick species. However, there is little prevalence data available in Thailand. Diseases presentation of blood parasites infection is similar, but the treatment of each species is different. Current diagnosis mainly relies on microscopic examination of a stained blood smear, which has low sensitivity. Therefore, accurate diagnosis is important. This study aims to evaluate the efficacy of the conventional polymerase chain reaction (PCR) method and routine blood smears in the detection of four blood parasites species in dogs from Buriram Province, Thailand.

**Materials and Methods:**

In total, 49 EDTA-blood samples were collected from dogs in Buriram Province, Thailand. Blood parasite infection was compared using the Giemsa-stained blood smear technique to identify the parasite under a 100× oil immersion with PCR amplification of the 18S rDNA gene of *B. canis* and *H. canis* and the 16S rDNA gene of *E. canis* and *A. platys*.

**Results:**

Only one dog out of 49 was positive for *H. canis* based on microscopic examination whereas the PCR results showed that 2.04% (1/49), 4.08% (2/49), 36.73% (18/49), and 30.61% (15/49) of dogs were positive for *B. canis, H. canis, E. canis*, and *A. platys*, respectively. Moreover, coinfection was found in 16.33% (8/49) of dogs.

**Conclusion:**

This study is the first report to demonstrate the molecular prevalence of blood parasites in domestic dogs in Buriram Province. The results indicated that the PCR method exhibited much higher sensitivity and reliability for blood parasites diagnosis in dogs. Therefore, our data support serious concern regarding the diagnostic technique used in routine blood testing and also provide prevalence data for the management and control of blood parasites in this area.

## Introduction

Blood parasites infection is an important health problem causing morbidity and mortality in dogs worldwide [1-7]. In Thailand, dog blood parasites have spread throughout the country, with the four known types of vector-borne parasites being *Babesia* spp.*, Hepatozoon canis*, *Ehrlichia canis*, and *Anaplasma platys* [3,8-11]. *Babesia canis* and *H. canis* are intracellular protozoa belonging to the phylum Apicomplexa while *E. canis* and *A. platys* are Gram-negative, obligate intracellular organisms belonging to the Order Rickettsiales. These four parasites share the common vector, the brown dog tick (*Rhipicephalus sanguineus*) which is the most common tick species found in dogs in Thailand [12]. Dogs generally get infected through infected tick bites in the case of *B. canis*, *E. canis*, and *A. platys* or through ingestion of the infected tick in the case of *H. canis*. Blood parasites are also transmitted to another dog by blood transfusion [13,14]. From previous studies in Thailand, the prevalence of *Babesia* spp. infections were 6.3%, 9.4%, and 19.5% in Maha Sarakham, Songkhla, and Khon Kaen Provinces, respectively [3,8,9]. The prevalence of *Hepatozoon* spp. infections were 10.1%, 18.8%, and 11.4% in Maha Sarakham, Songkhla, and Bangkok Provinces, respectively [3,8,11]. The prevalence of *E. canis* was 21.5%, 3.9%, and 1.3% in Maha Sarakham, Songkhla, and Khon Kaen Provinces, respectively [3,8,9], and *A. platys* infection has been reported only in Songkhla Province with 4.4% prevalence [8]. Moreover, coinfection between *B. canis* and *E. canis* has been reported in 2.5% of dogs in Maha Sarakham Province [3].

Dog blood parasites cause similar disease presentations ranging from subclinical to severe pathology involving blood cells and multiple organs [1,2,4,15-18]. Common symptoms include fever, hemolytic anemia, thrombocytopenia, splenomegaly, and organ dysfunction [18,19]. Coinfection with other blood parasites has clinical importance because it complicates diagnoses, exacerbates clinical signs, reduces the effectiveness of treatment, and can worsen the prognosis [20,21]. Nowadays, the diagnosis of dog blood parasites in Thailand is mainly based on microscopic examination of blood smears. However, this technique has low sensitivity, particularly in low parasitemia cases, and it also requires an experienced examiner [22-24]. A serological diagnostic kit has been used for diagnosis of *Ehrlichia* spp. and *Anaplasma* spp. in Thailand, but the major limitation of a serological test is that it cannot discriminate between recent and present infection [25]. Polymerase chain reaction (PCR) is a widely used molecular technique to confirm blood parasite infection because it has high sensitivity and specificity [3,9,10,26,27].

In Thailand, little epidemiological data are available, and no comparison between PCR detection and blood smear has been reported. Therefore, we aimed to identify the efficacy of the conventional PCR compared with blood smear technique to survey the prevalence of *B. canis, H. canis*, *E. canis*, and *A. platys* in domestic dogs from Prakhon Chai district, Buriram Province, Thailand.

## Materials and Methods

### Ethical approval

This study was approved by the Animal Ethics Committee of the Faculty of Veterinary Technology, Kasetsart University, Thailand (ACKU61-VTN-004).

### Dog blood sample collection

Forty-nine ethylenediaminetetraacetic acid (EDTA) blood samples were randomly collected from domestic dogs in Prakhon Chai district, Buriram Province, Thailand. 21 (42.86%) were males and 28 (57.14%) were females. All dog had no obvious clinical signs of blood parasite infections at the time of blood collection such as fever, lethargy, emaciation, or pale mucous membrane. EDTA blood samples were stored at −20°C until extraction of DNA. The positive samples for *B. canis, H. canis*, *E. canis*, and *A. platys* infection confirmed by blood smear were used as positive controls. All positive controls were obtained from the Veterinary Teaching Hospital, Faculty of Veterinary Medicine, Khon Kaen University, Thailand.

### Blood smears

Blood smears were performed on the same day as blood collection. After being air-dried, the thin smears were fixed in methanol and were stained for 20 min in Giemsa stain. The slides were then rinsed with tap water, air-dried, and examined using a 100× oil immersion objective lens. Areas that were well-stained, free of stain precipitate, and well-populated were selected and examined on at least 100 fields of view.

### DNA extraction

DNA was extracted from 200 µl of whole blood using FavorPrep Blood Genomic DNA Extraction Mini Kit (Favorgen Biotech Corp.) according to the manufacturer’s protocol. Briefly, 20 µl proteinase K and 200 µl FABG buffer were added to the samples, which were then mixed and incubated at 60°C for 15 min to lyse the cells. The tube was centrifuged to remove drops on the cap and then 200 µl absolute ethanol was added to the sample. After mixing thoroughly using pulse-vortexing for 10 s and briefly spinning the tube to remove drops, the mixture was placed in a FABG Mini Column, washed, and eluted for DNA solution.

### Primer design and PCR amplification

All primers were designed using the Primer3 program online tool available at http://bioinfo.ut.ee/primer3-0.4.0/to flank around the selected DNA region. For the 18S rDNA gene, three DNA sequences of *B. canis vogeli* (Acc. No. AY072925.1), *B. canis canis* (Acc. No. AY072926.1), and *H. canis* (Acc. No. DQ439543.1) were retrieved from the NCBI nucleotide database and used as input for DNA alignment with Clustal Omega which was available online at https://www.ebi.ac.uk/Tools/msa/clustalo/. The polymorphic regions were used to target DNA priming. For the 16S rDNA gene, two sequences, *E. canis* (EU178797.1) and *A. platys* (LC269820.1), were downloaded from the same database and the same strategy was adopted as used in the 18S rDNA design. All designed primers used in the experiment are listed in Table-1.

**Table-1:** Targeted genes and list of new primers used in this study.

Gene	Primer name	Sequence (5’>3’)	Product size
18S rDNA	BH18SF BH18SR	AATTGGAGGGCAAGTCTGGT TGCTTTCGCAGTAGTTYGTC	356 bp (for *Babesia canis vogeli*) 357 bp (for *Babesia canis canis*) 409 bp (for *Hepatozoon canis*)
16S rDNA	16SFEhr 16SFAna 16SR	CTGCTAGACTAGAGGTCGAA GGGCATGTAGGCGGTTCG CTCATCGTTTACAGCGTGGA	181 bp (for *Ehrlichia canis*) 250 bp (for *Anaplasma platys*)

PCR was performed using the newly designed primers for amplification of the 18S rDNA sequences of *B. canis* and *H. canis* and the 16S rDNA sequences of *E. canis* and *A. platys*. Positive samples of *B. canis, H. canis*, *E. canis*, and *A. platys* were used as positive controls. The PCR reactions were performed in a 50 µl reaction composed of 1× DreamTaq Green buffer (Thermo Scientific), 0.2 mM dNTP each, 1 µM of each DNA primer, 100 ng of DNA template, 1.25 units of DreamTaq DNA polymerase (Thermo Scientific), and ultrapure sterile water up to 50 µl. The amplification procedure consisted of the following steps: 2 min at 95°C for initial denaturation, denaturation for 30 s at 95°C, annealing for 30 s at 55°C, and extension for 30 s at 72°C. Steps 2-4 were repeated for another 39 cycles and followed by a final extension for 5 min at 72°C. The PCR products were identified using 1.0% agarose gel electrophoresis stained with SYBR Safe DNA gel stain (Thermo Scientific) to verify the amplicon size under UV light. 19 PCR products were randomly selected from the positive PCR products and subjected to nucleotide sequencing. All DNA sequences were later aligned to either 18S rDNA or 16S rDNA templates to confirm the identity of the amplified fragment.

### Statistical analysis

SPSS version 25.0 was used for statistical analysis. Agreement between the blood smear and PCR techniques was determined using the Kappa (K) test.

## Results

### Primer design

**Figure-1:**
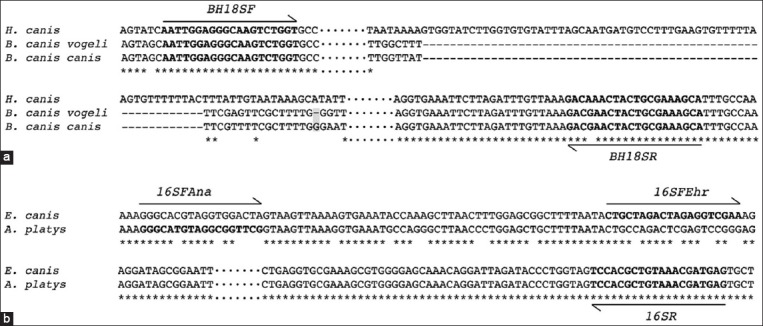
Simplified DNA alignment showing polymorphic regions targeted in primer design of (a) 18S rDNA and (b) 16S rDNA. Dot symbols show omitted regions that may not be involved in the result interpretation and discussion. Shaded area shows the 1-bp insertion between *Babesia canis* subspecies.

Both 18S rDNA and 16S rDNA genes serve as a housekeeping gene and have a high copy number in their genomes of eukaryotes and prokaryotes, respectively, allowing us to have sufficient amount of DNA template for PCR amplification in light infection. The DNA alignment of those genes using Clustal Omega revealed the polymorphic regions among the same genes. These regions showed promising capacity in the primer design for use as a molecular marker separating the species-specific infection. For the 18S primer, the forward and reverse primers were designed at the same position flanking the insertion region of *H. canis* and making the amplicon size larger than the fragment from *B. canis*. It should be noted that the DNA alignment between *B. canis canis* and *B. canis vogeli* revealed a slightly different DNA sequence with a 1-bp missing base in *B. canis vogeli* (shaded area in Figure-1a) making subspecies identification possible using some molecular detection techniques such as DNA sequencing or single-strand conformation polymorphism.

In the 16S rDNA primer design, the forward primers were designed to contain a species-specific selection using the 3’ end priming site. Since only the 3’end priming of the primer would play an important role in the DNA extension process, the 16SFAna primer was designed to have a 4-bp specific binding site at the 3’ end of the *A. platys* template, whereas the 16SFEhr primer contained a 2-bp specific binding site at the 3’ end of the *E. canis* template (Figure-1b). These specificities would allow the successful amplification by certain parasite templates. A reverse primer was used for both species priming.

### Blood smear and PCR prevalence rates

**Figure-2:**
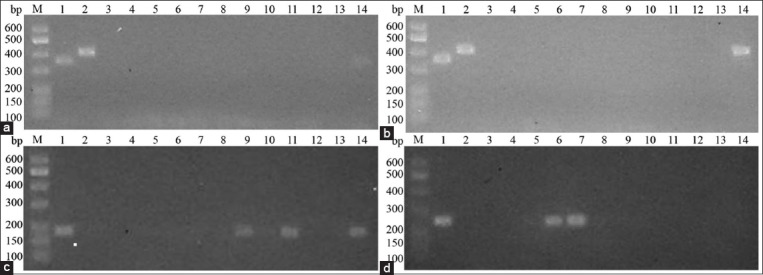
Example of polymerase chain reaction gels of (a) *Babesia canis* amplicons at 356 bp (b) *Hepatozoon canis* amplicons at 409 bp (a and b, Lane 1 = *B. canis* positive control, Lane 2 = *H. canis* positive control, Lanes 3-14 = samples). (c) Example of *Ehrlichia canis* amplicons at 181 bp and (d) *Anaplasma platys* amplicons at 250 bp (c and d, Lane 1 = positive control, Lanes 2-14 = samples). Corresponding lane numbers between figures (a-d) are not related to the same sample.

Out of the 49 dogs, only 2.04% (1 dog) was observed as positive for *H. canis* infection, which was identified using Giemsa-stained blood smear examination under a light microscope. On the other hand, the PCR results showed that 57.14% (28/49) of dogs were positive for at least one blood parasite infection and the greatest prevalence was with *E. canis* corresponding to 36.73% (18/49) with lower values for *A. platys, H. canis, and B. canis* infection with the prevalence scores of 30.61% (15/49), 4.08% (2/49), and 2.04% (1/49) of dogs, respectively. Examples of successfully amplified DNA bands are shown in Figure-2a-d, respectively, at 356 bp for *B. canis*, 409 bp for *H. canis*, 181 bp for *E. canis*, and 250 bp for *A. platys*. In addition, it should be noted that multiple infections were found in 16.33% (8/49) of dogs. Seven dogs were coinfected with *E. canis* and *A. platys* and one dog was coinfected with *E. canis* and *H. canis*. The results of the prevalence of each parasite are shown in Table-2. Finally, there were 2.04%, 2.04%, 36.73%, and 30.61% false-negative results detected through microscopic examination of *B. canis, H. canis, E. canis*, and *A. platys*, respectively.

**Table-2:** Comparison between blood smear and PCR techniques regarding the prevalence of tick-borne pathogens infection among dogs.

Pathogen/techniques	Blood smear		PCR	
	
	% Positive (number)	% Negative (number)	% Positive (number)	% Negative (number)
*B. canis vogeli*	0 (0)	100 (49)	2.04 (1)	97.96 (48)
*H. canis*	2.04 (1)	97.96 (48)	4.08 (2)	95.92 (47)
*E. canis*	0 (0)	100 (49)	36.73 (18)	63.27 (31)
*A. platys*	0 (0)	100 (49)	30.61 (15)	69.39 (34)

*B. canis vogeli=Babesia canis vogeli*, *H. canis=Hepatozoon canis, E. canis=Ehrlichia canis, A. platys=Anaplasma platys*, PCR=Polymerase chain reaction

## Discussion

Although microscopic examination of Giemsa-stained blood smear is practical and routinely used to detect dog blood parasites infection, the tests do have recognized limitations and require high parasitemia [9,28]. The current study revealed that false-negative results were as high as 36.73% detected through microscopy. Dogs with low numbers of blood parasite infection had few infected cells which hampered the microscopic examination [29]. No dogs in this study have any significant signs of infection. Thus, molecular detection produced better results.

Our results supported the hypothesis that dog blood parasites remain a health problem in dogs in Thailand. We demonstrated that dogs were positive with all four types of blood parasites using the PCR technique. The prevalence of *B. canis* infection (2.01%) was lower than reported in previous studies (6.3% and 19.5%) in the same region of Northeastern Thailand [3,9]. Furthermore, a lower prevalence was found in *H. canis* infections (4.08%) compared with 10.1%, 18.8%, and 11.4% in previous studies [3,8,11]. Interestingly, the molecular prevalence of *E. canis* infection in dogs with owners in this study (36.73%) was higher than the prevalence obtained from stray dogs from Maha Sarakham Province (21.5%) [3] and domestic dogs from Khon Kaen Province (3%) [9]. Moreover, the prevalence of *A. platys* (30.61%) in the present study was higher than that in a previous study in Songkhla Province (4.4%), Southern Thailand [8]. Most coinfection was found with *E. canis* and *A. platys*, which may lead to more severe anemia than from a single infection [21]. *H. canis* and *E. canis* coinfection was found that agreed with the findings presented in a previous report in Khon Kaen Province [9].

Although PCR has higher sensitivity and specificity than blood smear, the cost and time may remain a major limitation for the use of PCR to detect all common blood parasites. We try to improve the cost-effectiveness of PCR by designing new primers. We successfully detected *B. canis* and *H. canis* using the developed single primer pair providing easy PCR reaction for *B. canis* and *H. canis* in a single tube. This strategy could be applied to the detection of *E. canis* and *A. platys* since a reaction required the unique forward primer to specifically bind at the 3’ end of the priming site leading to assessment in one reaction tube. Moreover, all primers used in this study were optimized for the same annealing temperature of 55°C allowing all PCR reactions to be simultaneously performed in the same run on the machine. This study used 40 cycles of PCR amplification for the detection of four common blood parasites compared to 35 cycles in other studies [3,5,6] because a faint band was observed when 35 cycles used.

Based on our results, there is a very serious quality problem with blood smear detection. Therefore, we recommend that the diagnosis of blood parasites in dogs should not be based on the routine blood smear technique alone. PCR is endorsed for routine blood parasites diagnosis and also suggested for the screening of blood donors in animal blood banks to prevent blood-borne pathogen transmission [14,30]. However, PCR is not suitable for monitoring the response to treatment because the result may be falsely positive for several days after parasites eliminated [31]. This study also suggested that there is an underestimation of the blood parasite prevalence in dogs in Thailand. Our results may provide valuable data for eradication, prevention, and control of dog blood parasites in Buriram Province and other parts of Thailand, which may be at risk of blood parasites infection.

## Conclusion

This study was the first to provide information on the molecular prevalence of four common dog blood parasites: *B. canis, H. canis*, *E. canis*, and *A. platys* using conventional PCR in domestic dogs in Buriram Province, Northeastern Thailand. The results suggested that PCR is an effective method for definitive diagnosis of dog blood parasites infection, particularly in animal hospitals, blood banks and for an epidemiological study.

## Authors’ Contributions

RR designed and performed the experiments. CM and TM performed the experiment. RR, CM, and MS analyzed the data. PK collected the samples. RR and CM drafted the manuscript. All authors read and approved the final manuscript.
